# Buffer substitution in malaria rapid diagnostic tests causes false-positive results

**DOI:** 10.1186/1475-2875-9-215

**Published:** 2010-07-22

**Authors:** Philippe Gillet, Marcella Mori, Jef Van den Ende, Jan Jacobs

**Affiliations:** 1 Department of Clinical Sciences, Institute of Tropical Medicine (ITM), Antwerp, Belgium; 2 Department of Medical Microbiology, Faculty of Health, Medicine and Life Sciences (FHML), Maastricht, The Netherlands

## Abstract

**Background:**

Malaria rapid diagnostic tests (RDTs) are kits that generally include 20 to 25 test strips or cassettes, but only a single buffer vial. In field settings, laboratory staff occasionally uses saline, distilled water (liquids for parenteral drugs dilution) or tap water as substitutes for the RDT kit's buffer to compensate for the loss of a diluent bottle. The present study assessed the effect of buffer substitution on the RDT results.

**Methods:**

Twenty-seven RDT brands were run with EDTA-blood samples of five malaria-free subjects, who were negative for rheumatoid factor and antinuclear antibodies. Saline, distilled water and tap water were used as substitute liquids. RDTs were also run with distilled water, without adding blood. Results were compared to those obtained with the RDT kit's buffer and *Plasmodium *positive samples.

**Results:**

Only eight cassettes (in four RDT brands) showed no control line and were considered invalid. Visible test lines occurred for at least one malaria-free sample and one of the substitutes in 20/27 (74%) RDT brands (saline: n = 16; distilled water: n = 17; and tap water: n = 20), and in 15 RDTs which were run with distilled water only. They occurred for all *Plasmodium *antigens and RDT formats (two-, three- and four-band RDTs). Clearance of the background of the strip was excellent except for saline. The aspects (colour, intensity and crispness) of the control and the false-positive test lines were similar to those obtained with the RDT kits' buffer and *Plasmodium *positive samples.

**Conclusion:**

Replacement of the RDT kit's dedicated buffer by saline, distilled water and tap water can cause false-positive test results.

## Background

### The use of malaria RDTs is rapidly expanding

Malaria rapid diagnostic tests (RDTs) detect *Plasmodium *parasites in blood by an antibody-antigen reaction on a nitrocellulose strip. Reactions are visible as cherry-red lines. Two-band RDTs are mostly designed to detect *Plasmodium falciparum*; they display a control line and a test line, which targets either histidine-rich protein-2 (HRP-2) or *P*. *falciparum*-specific parasite lactate dehydrogenase (Pf-pLDH). Three- and four-band RDTs display a control line and two or three test lines, one targeting *P*. *falciparum *specific antigen, another line targeting antigens common to the four species, such as pan-*Plasmodium*-specific lactate parasite dehydrogenase (pan-pLDH) or aldolase, and, in case of the four band RDTs, a third line which targets *Plasmodium *vivax-specific pLDH (Pv-pLDH).

RDTs are currently rolled out by national malaria control programmes in endemic settings as a tool for parasite based diagnosis in the scope of artemisinin-based combination therapy (ACT) [1]. In 2007, more than 70,000,000 tests were performed [2]. During the last few years, RDTs have technically improved and so-called one-step tests have replaced the older multistep tests. However, despite their apparent simplicity, they are not completely fail-proof [3,4].

### The single vial of buffer in RDT kits may cause problems of availability

Most malaria RDTs are marketed as kits that include material for 20 to 25 tests, such as lancets for finger pricking, test strips (available as dipstick, cassette or card box formats), transfer devices (pipettes, straws, capillaries or loops) and the buffer. Cassettes are usually individually packaged, and the number of lancets and transfer devices match the number of cassettes. All RDTs need a buffer to lyse the blood and to allow capillary flow along the nitrocellulose strip. Mostly, this buffer is supplied in a single bottle or dropper vial.

During field visits (particularly in Africa, ITM teams repeatedly observe availability problems of buffer vial: for instance, some cassettes were sent for testing in the ward, but the buffer vial did not return. To compensate, laboratory technicians took either a buffer vial from another kit (sometimes a kit of another brand), or used saline, distilled water (liquids for parenteral drugs dilution) and occasionally tap water as substitute liquids. Apparently, this substitution for the buffer seemed not to cause too much interference, as in all observed cases, there was enough background clearance and both control line and test lines were clearly distinguishable.

This phenomenon was also noted during a practical teaching session at the Institute of Tropical Medicine (ITM): students and staff were astonished to observe a visible HRP-2 line when the blood of one of the present authors (PG) was tested with a two-band RDT which was run with distilled water. PG had no symptoms of malaria, nor did he suffer from malaria in the recent past. When performing the RDT with the kit's dedicated buffer, there was no HRP-2 line visible. The HRP-2 line appeared upon retesting with distilled water and also when using saline and tap water as substitute liquids. As false-positive test line seemed to be the explanation, it was decided to explore this phenomenon.

## Methods

### Samples of healthy subjects and Plasmodium positive samples

EDTA-blood samples from five healthy subjects with no recent history of malaria were used. For all samples, the diagnosis of malaria was excluded by microscopy and species-specific PCR as previously described [5,6]. The presence of known causes of false positive RDT results such as the rheumatoid factor or antinuclear antibodies was ruled out [7-9] and none of the subjects had been manipulating mice during the past ten years, thereby reducing the probability of false positive results due to anti-mouse antibodies [10]. For most experiments, fresh samples were used; samples stored at -70°C were used in the case of delays of delivering of the RDTs. For comparison, all RDTs were run with their kit's dedicated buffer and two clinical samples, one infected with *P. falciparum *and another with *P. vivax*, at parasite densities of 36,140 and 3,600/μl respectively.

### Choice of malaria RDTs

Malaria RDTs marketed as cassettes and folded card box were selected, and RDT brands commonly used in field settings were included. CE marking and FDA approval of the RDTs were recorded, as well as their presence on the World Health Organization (WHO) lists of RDT manufacturers and distributors complying with ISO13485:2003 or US FDA 21 CFR 820 production norms and their evaluation by the World Health Organization/Foundation for Innovative New Diagnostics (WHO/FIND) [11,12]. RDTs that were not on the local (Belgian) market were directly ordered from the manufacturer. In view of the wide lot-to-lot variations and the ever changing composition of RDTs, it was decided not to display the individual RDT brand and kit names, in line with previous comparative studies assessing characteristics of RDTs [13,14].

### RDT test procedures

All RDT kits were used within their expiry date and had been stored at room temperature (maximum 25°C) before analysis. RDTs were assessed in the same run with the five subjects' blood and the following buffers: RDT kit's dedicated buffer, distilled water (Denolin, Brussels, Belgium), saline (NaCl 0.9%, Qualiphar, Bornem Belgium) and tap water from local supply. For each RDT and substitute liquid, the blood samples of all five subjects were used except when a particular RDT kit was finished. In addition, tests were run in the absence of blood, with distilled water as the substitute liquid.

All tests were performed according to the instructions of the manufacturer, except that samples were loaded with a pipette (Finnpipette, Helsinki, Finland) instead of the transfer device supplied by the manufacturer. Readings were performed by three readers at daylight assisted by a standard electric bulb, and within and not beyond the prescribed delay after application of the sample and buffer.

### Interpretation of results

In case the control line did not appear, the result was considered as invalid and the test was repeated. RDT test lines were interpreted according to the manufacturers' instructions. In addition, test line intensities were scored into five categories: none (no line visible), faint (barely visible line), weak (paler than the control line), medium (equal to the control line) or strong (stronger than the control line) [6]. Observers were blinded to each others' reading. The results of the readings considered were based on consensus agreement [15].

The appearance, shape and crispness of the control and test lines and the clearance of the background were compared with those obtained with the RDT kit's dedicated buffer and the two *Plasmodium*-positive samples. Visible test lines observed in the malaria free subjects' samples will be further referred to as "false-positive test lines".

### Inter-observer agreement and reproducibility

Inter-reader reliability was assessed and expressed as percentage agreements for all three readers and kappa values for each pair of readers. To assess test reproducibility, a sample from one subject was tested upon five occasions for all RDT brands and the three substitute liquids. Two RDT brands were not included in the reproducibility assessment because of shortage of tests.

### Ethical review

The study was approved by the Institutional Review Board of ITM and by the Ethical Committee of Antwerp University, Belgium.

## Results

### Selection of malaria rapid diagnostic kits

Thirty different RDT brands were selected. As two brands from the same manufacturer showed bad clearance of the background upon testing with *Plasmodium*-positive samples and another brand had a very low specificity, they were not included in the study. The final panel consisted of 27 brands (26 cassette and 1 card box format). Eleven (40.7%) of them had CE-mark compliance, one was approved by the U.S. Food and Drug Administration, 22 (81.5%) were included in the WHO list of RDTs adequate evidence of good manufacturing practice (GMP), 22 (81.5%) were evaluated by the WHO/FIND [11,12], and 9 (30%) are included in the list of malaria RDTs eligible for procurement by WHO [16]. The 27 RDT brands comprised two-, three- and four-band RDTs (Table 1) and all *Plasmodium *target antigens (HRP-2, Pf-pLDH, Pv-pLDH, pan-pLDH and aldolase) (Table 2).

**Table 1:** Numbers of RDT brands showing false positive test lines when run with blood of malaria free subjects and substitute liquids

RDT format	Numbers of different RDT brands assessed	Numbers of RDTs brands showing false positive test lines when run with substitute liquid*			
		
		Saline	Distilled water	Tap water	Any substitute liquid
Two band	6	3 (1)	3 (1)	4 (1)	4 (1)
Three band	17	12 (9)	11 (10)	12 (9)	12 (10)
Four band	4	1 (0)	3 (2)	4 (2)	4 (2)
Total	27	16 (10)	17 (13)	20 (12)	20 (13)

*Numbers refer to the different RDT brands for which false positive test lines were visible in at least one sample. Between brackets: numbers of RDT brands for which false positive lines were visible in the samples of at least three subjects.

**Table 2:** Numbers of false-positive test lines for the different *Plasmodium *antigens of the RDTs when run with blood of malaria free subjects and substitute liquids

RDT target antigen	Numbers of RDT brands (n = 27) detecting the target	Total numbers of tests performed with each substitute*	Numbers of false positive test lines when run with substitute liquid		
		
			Saline	Distilled water	Tap water
HRP-2	21	98	22	28	36
Pf-pLDH	5	25	8	9	9
Pv-pLDH	9	45	12	21	20
Pan-pLDH	14	70	30	25	33
Aldolase	3	11	1	5	5

*Each RDT brand was assessed with blood of five malaria-free subjects except one HRP-2-based kit and one HRP-2/aldolase-based kit that were assessed with two and one samples respectively.

### Results for the RDTs when run with samples of malaria free subjects and substitute liquids

When run with their kit's buffer, none of the RDT brands tested positive with any of the samples of the five malaria free subjects. Likewise, when tested with the two *Plasmodium *positive blood samples and the RDT kit's buffer, the expected test line results were observed.

When tested with a substitute liquid, there were eight invalid test results; they neither showed a control line upon repetition. Five of them occurred in a single RDT brand when assessed with distilled water. False-positive test lines were visible for at least one sample and substitute liquid in 20/27 (74%) RDT brands, at the following frequencies: saline: n = 16, distilled water n = 17 and tap water: n = 20 (Table 1). They occurred randomly among the samples of the five malaria free subjects, and occurred in at least three of them. For 10 RDT brands, all five blood samples tested positive with at least one of the substitute liquids (saline: n = 4, distilled water: n = 5, tap water: n = 7). Two-, three- and four-band RDTs were all affected, as well as all *Plasmodium *target antigens (Table 2).

**Figure 1:**
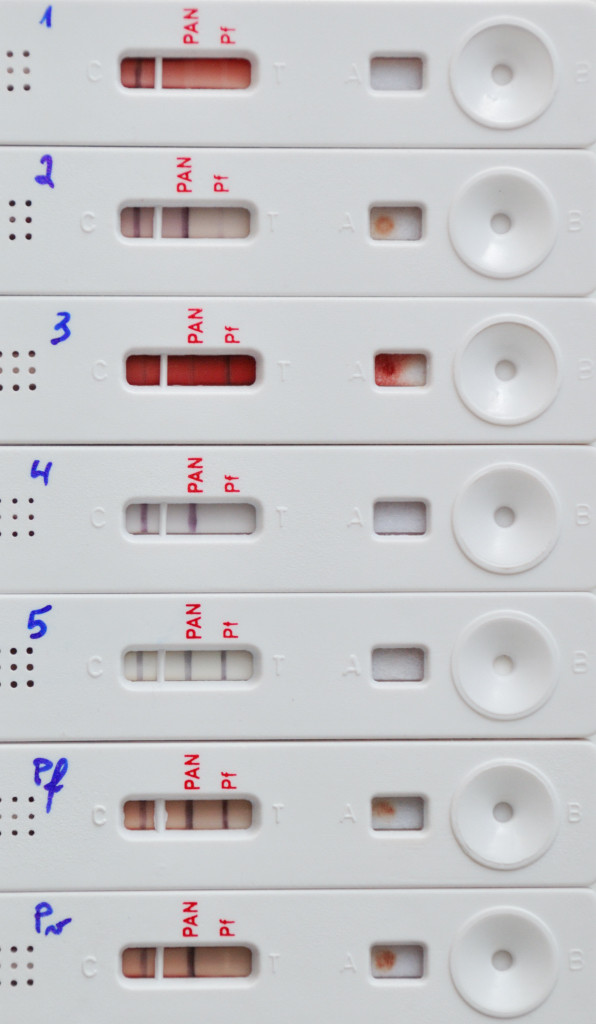
**Example of buffer substitution's effect on a three-band RDT**. Three-band RDT cassettes run with blood of a malaria free subject and the RDT kit's dedicated buffer (1), injection water (2), saline (3), tap water (4) and when run with injection water in absence of blood sample (5). Cassettes "Pf" and "Pv" refer to *Plasmodium falciparum or Plasmodium vivax *positive samples run with the RDT kit's dedicated buffer. All cassettes show regular control lines, cassette 1 shows the expected result (no test line visible), cassettes 2, 3 and 5 show false positive pan-pLDH and Pf-HRP2 lines, cassette 4 shows a false positive pan-pLDH line, and cassettes "Pf" and "Pv" show the expected results (positive for pan-pLDH and Pf-HRP-2 lines or positive for pan-pLDH line respectively). In the cassette 3, the false positive reaction is partially masked by the background.

**Figure 2:**
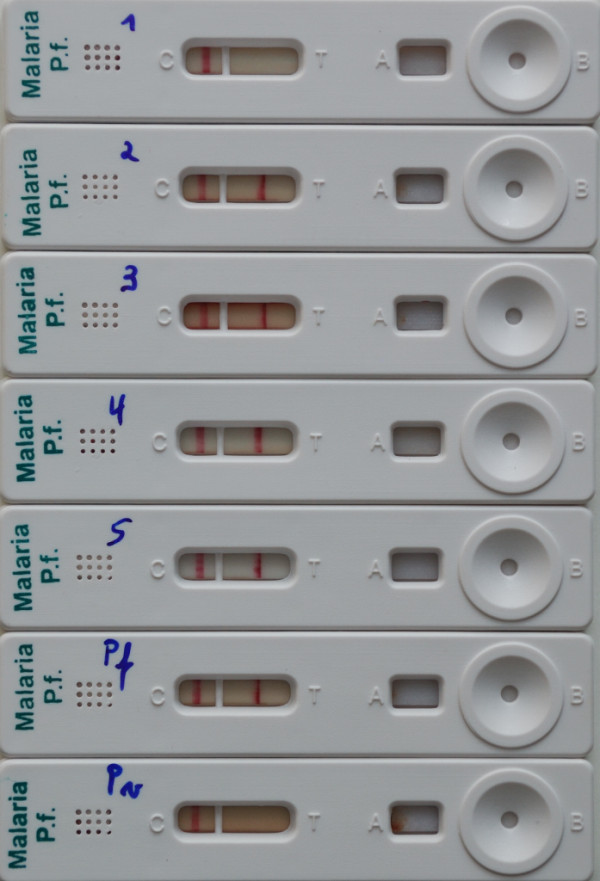
**Example of buffer substitution's effect on a two-band RDT**. Cassettes of a two-band RDT run with blood of a malaria free subject and the RDT kit's dedicated buffer (1), injection water (2), saline (3), tap water (4) and when run with injection water in absence of blood sample (5). Cassettes "Pf" and "Pv" refer to *Plasmodium falciparum *or *Plasmodium vivax *positive samples run with the RDT kit's dedicated buffer. All cassettes show regular control lines, cassette 1 shows the expected result (no test line visible), cassettes 2, 3, 4 and 5 show false positive Pf-HRP-2 line, cassettes "Pf" and "Pv" show the expected results (positive for Pf-HRP-2 line or no test line visible respectively).

For distilled water and tap water as the substitute liquid, the RDT strips with false-positive lines showed a clearance of background similar to those observed with the RDT kit's buffer. In the case of saline, the background was less clear. Overall, the colour and crispness of the control and false-positive test lines were similar to those obtained with the RDT kits' buffers and *Plasmodium *positive samples (Figure 1 and 2). The proportions of medium and strong line intensities for all substitute liquids combined were as follows: 26.7% (23/86) for HRP-2, 11.5% (3/26) for Pf-pLDH, 37.7% (20/53) for Pv-pLDH and 29.6% (26/88) for pan-pLDH. All false positive aldolase lines (n = 11) were either faint or weak. To the exception of better clearance of the background, the use of frozen sample did not influence the outcome of the results.

### Results for the RDTs when run with distilled water only

When tested with distilled water in the absence of blood, there was only a single invalid cassette, in which neither a control line appeared upon retesting. Control lines were observed in all remaining runs. Twenty-two false positive test lines were observed in 15 (55.6%) RDT brands and occurred in all *Plasmodium *targets (Table 3). Control and false positive test lines showed colour, thickness and crispness similar to those obtained with the RDT kits' buffers and *Plasmodium*-positive samples (Figure 1 and 2). The line intensities of false positives have however tended to be lower (Table 3).

**Table 3:** Numbers of false-positive test lines for the different *Plasmodium *antigens of the RDTs when run with distilled water in absence of blood sample

RDT target antigen	Numbers of RDT brands (n = 27) detecting the target	Numbers of false positive test lines	Numbers of false positive test lines according to test line intensity			
			
			Faint	Weak	Medium	Strong
HRP-2	21	11	2	5	3	1
Pf-pLDH	5	2	1	1	0	0
Pv-pLDH	9	1	1	0	0	0
Pan-pLDH	14	6	1	4	1	0
Aldolase	3	2	1	1	0	0

### Inter-reader reliability and reproducibility

The inter-observer agreement for positive and negative readings was high, with 96.4% overall agreement between the three observers and kappa values between 0.94-0.98 for each pair out of three observers. Inter-observer agreements were similar for all three substitute liquids. Discrepancies between line intensity readings were limited to one category of line intensity (*e.g.*, line intensity read as weak by reader one and as medium (but not strong) by reader two).

At reproducibility testing, false positive test lines occurred in at least four out of five runs in approximately half of the *Plasmodium *targets lines affected (40.0% (10/25), 51.9% (14/27) and 51.6% (16/31)) for saline, distilled water and tap water respectively). In terms of RDT brands, they occurred in at least four runs in eight, eleven and nine brands for saline, distilled water and tap water respectively.

### Differences between the different RDT brands

CE-mark and non-CE labelled RDT brands were equally affected by the buffer substitution (11 out of 20, versus four out of seven). In addition, there were 11/22 WHO-GMP listed RTD brands affected versus four out of five RDTs that were not listed, 12/22 FIND evaluated brands versus three out of seven, and five out of nine RDTs from the WHO procurement list affected versus 10/18. Although the false-positive test lines occurred randomly among the different RDT brands, there were certain RDT brands that were more affected than others.

## Discussion

This study shows that replacing malaria RDT kit's buffers with saline, distilled water and tap water resulted in false-positive test lines in the majority of brands assessed. All *Plasmodium *targets and RDT formats were affected, and the control and false positive test lines observed were similar to those obtained with *Plasmodium*-positive samples and the RDT kits' buffers. In addition, approximately half of the RDT brands showed false positive test lines when run with distilled water in the absence of blood.

Malaria RDTs detect *Plasmodium *antigens by antibody-antigen interactions on a nitrocellulose test strip. The patient's blood and several drops of buffer are applied to the sample pad of the strip. They are attracted by the capillary action of a soak pad at the other end of the strip and start to migrate. First, they pass the so-called conjugation pad, which contains a detection antibody targeted to a *Plasmodium *antigen, such as HRP-2, Pf-pLDH, Pv-pLDH, pan-pLDH and aldolase. This detection antibody is a mouse-antibody that is conjugated to a signal, mostly colloidal gold. If present in the sample, the *Plasmodium *antigen is bound to this detection antibody-conjugate. The antigen-antibody-conjugate complex migrates further across the strip until it is bound to a second antibody, the so-called capture antibody. This capture antibody reacts to another epitope of the *Plasmodium *target antigen. As the capture antibody is applied on a narrow section of the strip, the complex with the conjugated signal will be concentrated and becomes visible as a cherry-red coloured line. The excess of detection antibody-conjugate that was not bound by the antigen and the capture antibody moves further towards the soak pad until it is bound to a goat anti-mouse antibody, thereby generating a control line [10,17].

The present study has its limitations. First, there are currently more than 80 different RDT brands marketed [1], by consequence the results of the present panel of 27 brands should not be extrapolated to all RDTs. However, some out of these 80 brands are identical products marketed either as strips and cassette formats, of which the cassette form was tested in this study, given the fact that cassettes proved to be superior in terms of end-user performance [18-20]. In addition, representative RDT brands were elected with a majority of them included in the WHO-GMP list, evaluated by WHO/FIND [11,12] or eligible for procurement by WHO [16]. Another limitation is the fact that the presence of anti-mouse antibodies, which can potentially produce false positive test lines was not ruled out by serologic testing [10]. However, none of the subjects reported long-term contact with mice, and the results obtained with the RDT kits' buffer were invariably negative. The most prevalent causes of false positive test lines from the sample side were excluded (rheumatoid factor and anti-nuclear antibodies) and the recommended reading delays were carefully respected thereby avoiding the backflow phenomenon: backflow is reverse migration of the antibody-signal conjugate with nonspecific bindings at the test line site, it is cited as the most common cause of false-positive reactions in RDTs [10]. Finally the physicochemical causes of the false-positive test lines were not explored, partly because of technical limitations, partly because buffer compositions are often proprietary.

The presence of the control line is the result of the binding of the conjugated mouse-raised detection antibody to a goat-raised anti-mouse capture antibody: irrespective of the correct buffer there will be nearly always a control line visible as long as migration has been achieved. For the false-positive test lines, the situation is different. Their scattered distributions within the five malaria-free subjects and their moderate consistency upon retesting suggest nonspecific reactions, such as binding of the negatively charged colloidal gold conjugate to the positively charged capture antibodies. As the false-positive lines also occurred with distilled water in the absence of blood, a crucial role for the buffer is apparent. Apart from lysis of the red blood cells and allowing capillary migration of the sample along the strip, the buffer has other functions: it re-solubilizes blocking agents, such as detergents, polymers and proteins on the sample pad and the dried detection antibody-colloidal gold conjugate on the conjugate pad. Further, it ensures optimal pH and ionic strengths for the antigen-antibody reactions [10,17,21]. Substitution of the buffer may contribute to the non-specific bindings of the conjugate to the capture antibody in different ways: (i) less stringent pH and ionic strength conditions allowing non-specific bindings, (ii) inefficient solubilisation of blocking agents from the sample pad and (iii) a slower capillary flow rate which in turn decreases flushing of the non-specific bindings. Adding to this are mechanical issues: during application of the capture antibody, the dispensing pipette may emboss a groove in the membrane, with an additional decrease in capillary flow rate [21].

The high amount of false positive test lines with substitute liquids was unexpected and has, to the best of our knowledge, not yet been reported. The fact that the false positive test lines showed colour and crispness similar to those generated by *Plasmodium*-positive samples adds to the problem. In addition, their line intensities were comparable (albeit somewhat lower) to those observed in previous RDT evaluations at ITM [6,15,22-24] and among the affected RDT brands, there were two-band RDT brands that are currently widely deployed in endemic field settings.

Although the phenomenon of buffer substitution was observed in various places and on different occasions, the real extent of this phenomenon in the field is unknown. Studies assessing errors made by end-users do not mention it [20,25] but their designs (observations by checklists) were not adapted to assess incidental errors. It is tempting to speculate that at least some of the reported discrepancies between molecular tests and results from RDTs in field settings might be attributed to buffer substitution: as an example, Veron and Carme reported apparently false-positive RDT results for which they raised the possibility of incorrect performance [26].

False negative and false positive might delay/or exclude true diagnosis, with consequences that can go up to death for patients. The consequences of false-positive RDTs extend those of individual patient care and the non-justified prescription of ACT treatment: like other errors by end-users, poor performance of RDTs will erode the health care workers' confidence in RDT test results thereby hindering the implementation of RDTs in treatment algorithms and malaria control programs [2]. Furthermore, as the buffer helps to ensure optimal conditions of pH and ionic strength, there is also a concern of possible false-negative results. Although this was not addressed in the present study, anecdotal information from colleagues and alumni in the field indicated also false negative results in case of simple substitution of the RDT kit's buffer by a vial from another kit, even from the same brand but from a different lot. Therefore minimal changes of buffer composition seem to cause critical effects. Finally, one might question whether other rapid diagnostic tests such as those detecting HIV-antibodies also suffer from this phenomenon.

Prevention of buffer substitution can be addressed in several ways. From daily use and evaluations of RDTs, it is clear that some manufacturers already supply buffer vials with a volume in excess to the numbers of tests included. A solution could be the provision of more than one vial per kit, ideally (if costs are not too high), small plastic vials dedicated for each individual cassettes, as is already the case for individually wrapped packages for self-testing (example: CareStart^® ^Malaria, Single Kit, Access Bio Inc., New Jersey, USA).

Complementary, RDT package inserts should mention not to use any other liquids apart from the buffer supplied with the kit. Likewise, the generic job aids on malaria RDT designed by WHO [27] could include a comment on the use of the RDT kit's buffer.

The issue of buffer substitution should further be addressed in RDT instructions and trainings at all levels of health care organization. With respect to the organization of RDT performance by the end users among health care workers, it is recommended that use of the correct buffer should be supervised by a laboratory officer and that the RDT kits' content should not be split.

## Conclusions

In conclusion, buffer substitution in malaria RDTs causes false positive test lines in the majority of brands tested. Preventive measures in terms of product design, packaging, instructions manuals and trainings are needed to alert for this potential error.

## List of abbreviations

ACT: Atimisinin based combination therapy; CE: Conformité Européenne; EDTA: Ethylene diamine tetra-acetic acid; FDA: Food and drug administration; FIND: Foundation for Innovative New Diagnostics; GMP: Good manufacturing practice; HRP-2: Histidine-rich protein 2; ISO: International organization for standardization; ITM: Institute of Tropical Medicine; NaCl: Sodium chloride; *P: Plasmodium*; Pan-pLDH: pan *Plasmodium*-specific parasite lactate dehydrogenase; PCR: Polymerase chain reaction; Pf-pLDH: *Plasmodium falciparum-*specific parasite lactate dehydrogenase; pLDH: parasite lactate dehydrogenase; Pv-pLDH: *Plasmodium vivax*-specific parasite lactate dehydrogenase; RDT(s): Rapid diagnostic test(s); WHO: World Health Organization.

## Competing interests

The authors declare that they have no competing interests.

## Authors' contributions

PG and JJ designed the study protocol, MM and JVDE made substantial contributions to the concept and design of the study. PG and MM carried out the test evaluations. PG, MM and JJ analyzed and interpret the results and drafted the manuscript. All authors critically reviewed the manuscript and approved the final manuscript.
